# Acute exercise on memory function: open vs. closed skilled exercise

**DOI:** 10.34172/hpp.2020.20

**Published:** 2020-03-30

**Authors:** Justin Cantrelle, Grace Burnett, Paul D. Loprinzi

**Affiliations:** Exercise & Memory Laboratory, Department of Health, Exercise Science and Recreation Management, The University of Mississippi, University, MS 38677, USA

**Keywords:** Cognition, Metacognition, Walking, Running

## Abstract

**Background:** Previous studies suggest that acute exercise may improve memory function. Few studies, however, have investigated the differential effect of the acute exercise movement patterns on memory. Such an effect is plausible, as research demonstrates that open-skilled exercise (e.g.,racquetball) may have a greater effect on memory-related neurotrophins (e.g., brain-derived neurotrophic factors) when compared to closed-skilled exercise (e.g. treadmill exercise). A key distinction between open- and closed-skilled exercise is that open-skilled exercises are those that require an individual to react in a dynamic way to a changing, unpredictable environment. Our aim in this study was to assess wether retrospective and prospective memory are differentially influenced from open- and closed-skilled acute exercise.

**Methods:** A within-subject design was employed. Participants (M_age_ = 20.6 years; 69% female)completed two visits, in a counterbalanced order. The two experimental conditions included open-skilled acute exercise (racquetball) and closed-skilled acute exercise (treadmill exercise),each lasting 30-minute at 60% of heart rate reserve (HRR). During both experimental conditions,participants completed short- and long-term assessments of retrospective and prospective memory function. Retrospective memory was evaluated across multiple word-list trials (e.g.,Trials 1-6, 20-minute delay, 24-hour delay).

**Results:** No significant effect of exercise was found on prospective memory. For retrospective memory, there was a significant main effect for condition, F(1, 57) = 5.33, P = 0.02, η^2^ = 0.004,main effect for trial, F(4.12, 234.9) = 227.85, P < 0.001, η^2^ = 0.46, but no condition by trial interaction, F(4.63, 264.08) = 1.022, P = 0.40, η^2^ = 0.002.

**Conclusion:** Retrospective memory was greater after closed-skilled exercise (treadmill) when compared to open-skilled exercise (racquetball).

## Introduction

Emerging research demonstrates that acute exercise is associated with enhanced memory performance, typically assessed from word-list paradigms.^1-7^ Mechanisms of this potential effect are multifold, including, for example, exercise-induced neuronal excitability, transcription factor expression, and growth factor production.^8^ Regarding the latter, a key growth factor that may mediate the effects of acute exercise on memory is brain-derived growth factor (BDNF) production. This key protein plays a critical role in synaptic plasticity, as well as long-term potentiation, a key cellular correlate of memory function.^8^ Notably, acute exercise can upregulate BDNF levels.^9^ We have previously discussed the synthesis and regulation of BDNF, as well as the potential role through which BDNF may mediate the effects of acute exercise on memory.^10^ Although it is conceivable, from a mechanistic perspective, that BDNF may mediate this effect,^10^ actual experimental studies in humans have provided mixed findings regarding whether BDNF causally mediates the effects of acute exercise on memory.^11^

As recently suggested^4^ and demonstrated,^12^ the type of acute exercise, notably whether it is an open vs. closed skilled exercise, may have a differential effect on cognitive function. Open-skill exercises are those that require an individual to react in a dynamic way to a changing, unpredictable environment (e.g., badminton, racquetball).^13^ Exercises such as walking and running would be considered “closed-skill” exercises as the environment is relatively stable, predictable, and self-paced.^13^ Recent research demonstrates that, at the same given intensity (60% of heart rate reserve, HRR), open-skilled acute exercise (30-min bout) was more effective in enhancing BDNF and executive function when compared to closed-skill acute exercise.^14^ In the current study, we are going to extend this emerging question by examining whether open vs. closed-skilled acute exercise has a differential effect on memory function, which, to date, has yet to be examined in the literature. For a comprehensive assessment of memory, herein we assess both retrospective (recall of past events) and prospective memory (completion of a task to occur in the future). Notably, few studies have investigated the effect of acute exercise on prospective memory.^1,2,6,15,16^ and no study has compared the potential differential effects of open vs. closed skilled exercise on prospective memory.Thus, we chose to focus on both prospective and retrospective memory for two reasons: 1) this provides a more comprehensive assessment of memory, and 2) no study has evaluated the effects of acute exercise, when considering both open- and closed-skilled exercise, on either prospective or retrospective memory.

## Materials and Methods

### Study design

Written consent was provided from all participants, prior to participation. A within-subject design was employed. Participants completed two visits, in a counterbalanced order. The two experimental arms included open-skilled acute exercise (racquetball) and closed-skilled acute exercise (treadmill exercise). During both experimental conditions, participants completed short- and long-term assessments of memory function (both retr­ospective and prospective memory).

### Participants

Recruitment occurred via a convenience-based, non-probability sampling approach (classroom announcement and word-of-mouth). Participants included undergraduate and graduate students ­between the ages of 18 and 25 years.

Additionally, participants were excluded if they were taking any drugs that influenced the CNS or:

- Self-reported as a daily smoker^17,18^
- Self-reported being pregnant^19^
- Exercised within 5 hours of testing^20^
- Consumed caffeine within 3 hours of testing^21^
- Had a concussion or head trauma within the past 30 days^22^
- Took marijuana or other illegal drugs within the past 30 days^23^
- Were considered a daily alcohol user (>30 drinks/month for women; >60 drinks/month for men)^24^

### Exercise assessment

In a counterbalanced order, on separate visits, participants were instructed to engage in either a 30-minute bout of treadmill exercise or a 30-minute bout of racquetball. Both bouts of exercise lasted for 30 minutes and occurred at 60% of their HRR. Heart rate was monitored continuously (polar heart rate monitor) and recorded every 5-minutes. As needed, we naturally increased their heart rate by, for example, hitting the ball harder, that way they would have to run a bit harder/faster to get to the ball. We aimed to create a natural, typical racquetball experience.

Following the 30-minute bout of exercise at 60% of HRR, participants walked slowly (self-selected pace) for 5-minutes. Following this 5-minute cool-down period, participants rested (sat) quietly for 5 minutes before commencing the memory assessment.

### Memory assessment

Short-term and long-term memory (retrospective memory) was assessed using a word-list paradigm.^25^ Participants were asked to view and immediately recall a list of 15 words (List A) five times in a row (Trials 1-5). Each word, one at a time, was presented on a computer screen for 3 seconds. Participants then were asked to view and immediately recall a list of 15 new words (List B). Immediately following the recall of List B, participants were asked to recall the words from List A (Trial 6). Following Trial 6, there was a 20-minute delay, involving watching a video (self-selected either The Office of Big Bang Theory). Following this 20-minute delay, participants recalled as many words as possible from List A. Following this, participants returned to the laboratory for a 24-hour follow-up assessment of List A.

*Prospective memory*

To assess prospective memory, the RPA-ProMem test (Royal Prince Alfred Prospective Memory Test) was used.^16,26,27^ Specifically, we used Form 2 of the RPA-ProMem. In brief, participants completed two laboratory and two naturalistic prospective memory tasks, including both time-based and event-based tasks. The first laboratory prospective memory task (short-term time-based) involved having the participant inform the researcher what their last meal was at a particular point in time during the lab visit (i.e., approximately 20 minutes after they finished exercising). The researcher gave these instructions approximately 5 minutes after the bout of exercise and it was up to the participant to remember to complete this task. For the second laboratory prospective memory task (short-term event-based), the researcher indicated that they would like to borrow something from the participant (e.g., phone or wallet), and when the alarm (the researcher’s phone alarm) goes off in the lab, the participant is to remind the researcher to give back the personal object.

One of the naturalistic prospective memory tasks (long-term event-based) included the participant texting the researcher on the phone when they got home or when eating dinner (whichever came first). The second naturalistic prospective memory task (long-term time-based) included the participant returning a piece of paper to the researcher during the 24-hour follow-up assessment, in which they were to write down the weather of that day (e.g., rainy, sunny, windy) or the high/low temperature that day (depending on the experimental condition).

For each of the 4 components (short-term time-based; short-term event-based; long-term time-based; and long-term event-based), participants were given a score between 0 and 3 (based on whether the task was completed correctly and on-time). For example, for the long-term time-based task, they received 3-points if they returned the piece of paper on the correct day with the correct information; 2-points if they returned the piece of paper on the incorrect day with the correct information; 2-points for the correct day but incorrect information; 1-point for the incorrect day and incorrect information; and 0-points if the piece of paper was not returned. Thus, the total points possible for the prospective memory task is 12, with a higher score indicating a better prospective memory performance.

### Protocol for visits

As stated, participants completed two main protocols, including 1) racquetball (open-skilled) exercise before the memory task, and 2) treadmill (closed-skilled) exercise before the memory task. These two main protocols occurred in a counterbalanced order. Details for these are as follows.

*Racquetball (open-skilled exercise)*

*Session 1*

- 30 minutes of racquetball exercise at 60% of HRR
- 5 minutes of self-selected walking pace for cool-down
- 5-minute seated rest
- Commence memory task
- Short-term time-based prospective memory (last meal eaten?)
- Seated rest for 20 minutes
- Delayed recall of memory task
  ▪ Short-term event-based prospective memory (alarm sounds)
- Long-term (time- and event-based) prospective memory task occurred between sessions 1 and 2.

*Session 2*

- Long-term (24-hour) recall of episodic memory

*Treadmill (closed-skilled exercise)*

*Session 1*

- 30 minutes of treadmill exercise at 60% of HRR
- 5 minutes of self-selected walking pace for cool-down
- 5-minute seated rest
- Commence memory task
  ▪ Short-term time-based prospective memory (last meal eaten?)
- Seated rest for 20 minutes
- Delayed recall of memory task
  ▪ Short-term event-based prospective memory (alarm sounds)
- Long-term (time- and event-based) prospective memory task occurred between sessions 1 and 2.

*Session 2*

 • Long-term (24-hour) recall of episodic memory

### Statistical analyses

All statistical analyses were computed in JASP (version .10; Amsterdam, The Netherlands). Descriptive statistics for continuous variables are reported as means and their associated standard deviation. A 2 (condition) × 8 (trials) repeated measures ANOVA was computed for the retrospective memory task. When violations to sphericity occurred, the Huynh-Feldt correction was applied. Paired samples *t* tests were computed for the prospective memory task (short- and long-term memory and time- and event-based). Effect size estimates (eta-squared for ANOVA or Cohen’s d for *t* tests) were calculated. Statistical significance was set at an alpha of 0.05.

## Results

Table 1 displays the characteristics of the sample. The sample included 58 participants (M_age_ = 20.6 years; 69% female).

**Figure 1:**
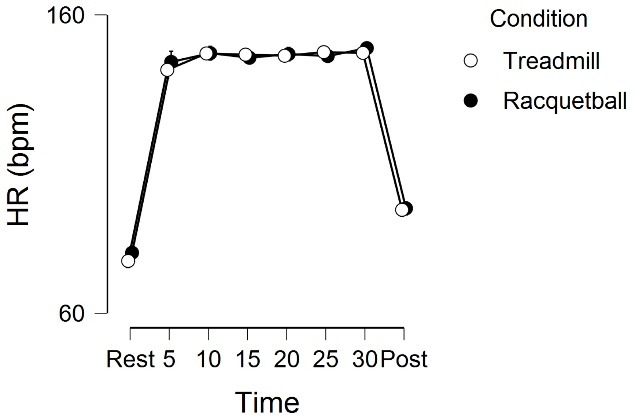
Heart rate responses across the 8 time periods (rest, 5-minute, 10-minute, etc) for the two experimental conditions. Error bars (minimally visible) represent 95% CI.

Figure 1 displays the physiological (heart rate) response to the exercise stimuli. There was a significant main effect for time, *F* (5.07, 289.3) = 1261.6, *P* < 0.001, η^2^ = 0.86, but no main effect for condition, *F* (1, 57) = 1.46, *P* = 0.23, η^2^ = 0.0001, or time by condition interaction, *F* (4.63, 263.7) = 1.27, *P* = 0.28, η^2^ = 0.001.

**Figure 2:**
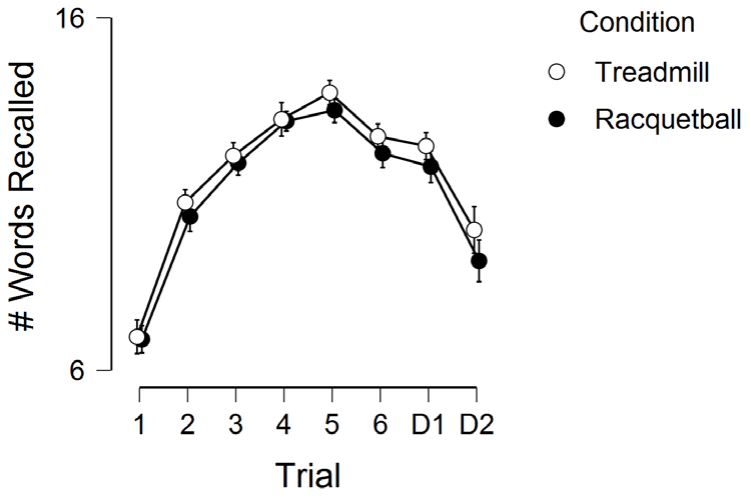
Word-list results across the experimental conditions. D1 and D2, respectively, represent the 20-min and 24-hr delay assessments. Error bars represent 95% CI.

Figure 2 displays the word-list results across the experimental conditions. There was a significant main effect for condition, *F* (1, 57) = 5.33, *P* = 0.02, η^2^ = 0.004, main effect for trial, *F* (4.12, 234.9) = 227.85, *P* < 0.001, η^2^ = 0.46, but no condition by trial interaction, *F* (4.63, 264.08) = 1.022, *P* = 0.40, η^2^ = 0.002. The main effect for condition appeared to be, in part, driven by differences occurring for Trial 5 (t(57) = 2.13, *P* = 0.03, d = 0.28, M_diff_ = 0.49), 20-minute delay (t(57) = 1.93, *P* = 0.05, d = 0.25, M_diff_ = 0.58), and the 24-hour delay (t(57) = 1.94, *P* = 0.05, d = 0.26, M_diff_ = 0.88), as there were no other differences for the other trials (*P* ’s > 0.10).

Table 2 displays prospective memory results. For all prospective memory outcomes, there were no statistically significant differences between the experimental conditions.

## Discussion

Previous studies have shown that acute exercise may, ptentially, improve retrospective memory. We,^28,29^ along with others,^30,31^ have summarized this effect elsewhere. Recently, we have also demonstrated that cognitive function may also be influenced by the mode of exercise.^12^ In a systematic review,^12^ we demonstrated that open-skilled exercise was superior in enhancing cognitive function when compared to closed-skill exercise. This was observed for observational and intervention studies, as well as for several cognitive outcomes, including memory. Notably, however, few experimental acute exercise studies were conducted.^14^ This served as the motivation for the present experiment, which was to evaluate whether acute open- or closed-skill exercise has a differential effect on memory function. Our two main findings are as follows, 1) closed-skilled exercise was more effective in improving retrospective memory in comparison with open-skilled exercise, and 2) there was no prospective memory difference between the two types of exercise. Given that this latter observation aligns with our past 5 experiments on prospective memory (i.e., no effect of acute exercise on prospective memory),^1,2,6,15,16^ the narrative that follows will just focus on our retrospective memory results.

We initially hypothesized that open-skilled exercise (racquetball) would be more effective in enhancing memory than closed-skilled exercise (treadmill). We anticipated that this would occur from greater cognitive demands as well as from potentially higher levels of neurotrophins that are likely to occur with open-skilled exercise.^12,14^ However, our retrospective memory results showed the opposite effect. Retrospective memory, particularly long-term retrospective memory, was greater after exercising on the treadmill as opposed to playing racquetball. Importantly, although this effect was statistically significant, the magnitude of this difference was minimal (Cohen’s d ranged from 0.25-0.28). Further, the mean difference between the experimental conditions for trial 5, 20-minute delay, and 24-hour delay, ranged from 0.49 to 0.88 words.

The present experiment did not collect any mechanistic data, whether it be cognitive, affective or neurophysiological, that may help to explain our unexpected findings. It seemed, however, that most of the participants were new to racquetball. If this is true, then perhaps the racquetball session induced greater cognitive load/demand when compared to treadmill exercise, which involves an unchanging environment requiring minimal cognitive engagement. Speculatively, perhaps exercise that induces greater cognitive demands may be more suitable for executive cognitions, which may require the utilization of multiple cognitive processes, such as inhibition, planning and reasoning. In contrast, perhaps exercise that induces greater cognitive demands has a less favorable effect for cognitions, such as retrospective memory, which may require fewer cognitive processes. As such, it would be worthwhile for future work to evaluate whether open- and closed-skilled acute exercise has a differential effect on distinct cognitive outcomes. Further, perhaps the affective response was different between the two conditions, which may have resulted in our unexpected findings. Although we do not know for certain, it seemed racquetball was new for most of the participants. Perhaps engaging in a new movement pattern, with poorer physical competency, altered their mood state, and in turn, influenced the degree to which they encoded the words for the retrospective memory task. Of course, this is pure speculation, but the possibility for affect to mediate the effects of acute exercise on memory is worth further critical reflection. Notably, however, other work, reviewed here,^28^ suggests that affective responses to exercise may not mediate the effects of exercise on memory.

A limitation of this experiment is not including a non-exercise control group. Similar to other related work on this topic,^14^ we intentionally chose not to include a control group because the aim of this experiment was not to evaluate whether acute exercise influences memory, as this has already been evaluated in numerous studies. The central focus of this experiment was comparing open- to closed-skilled exercise. However, we do acknowledge the benefit of including a non-exercise control group. Strengths of this study include the experimental design, study novelty, and relatively large sample for this type of experiment (see Figure 9 in Pontifex et al^32^).

In conclusion, the present experiment demonstrates that retrospective memory was greater after closed-skilled acute exercise (racquetball) when compared to open-skilled acute exercise (treadmill exercise). Exercise modality did not have a differential effect on prospective memory.

## Ethical approval

This study was approved by the ethics committee at the University of Mississippi (#19-046).

## Competing interests

The authors declare that they have no competing interests.

## Funding

No funding was used to prepare this manuscript.

## Authors’ contributions

All authors were involved in the conceptualization of the experiment. JC and GB collected the data. PL supervised the project and prepared the initial draft of the manuscript. All authors approved the final version of the manuscript.

**Table 1:** Sample characteristics

**Variable**	**Point Estimate**	**SD**
Age, mean years	20.7	1.4
Gender, % female	69.5	
Race-ethnicity, % White	84.7	
BMI, mean kg/m^2^	25.3	5.0
MVPA, mean min/wk	178.8	171.1

BMI, body mass index; MVPA, Moderate to vigorous physical activity (assessed from the Physical Activity Vital Signs survey).

**Table 2:** Prospective memory results across the experimental conditions

	**Treadmill**	**Racquetball**	**Test-Statistic**
Time-Based			
Short-term	2.33 (1.1)	2.20 (1.2)	t(57) = 0.77, *P* = 0.44, d = 0.10
Long-term	2.77 (1.5)	2.86 (1.1)	t(55) = 0.24, *P* = 0.80, d = 0.03
Event-based			
Short-term	2.41 (1.1)	2.41 (1.1)	t(57) = 0.001, *P* = 0.99, d = 0.001
Long-term	2.15 (1.2)	1.85 (1.3)	t(56) = 1.58, *P* = 0.11, d = 0.21
Overall	9.67 (3.0)	9.32 (3.0)	t(55) = 1.17, *P* = 0.24, d = 0.16
